# Winter fidelity, movements, and energy expenditure of Midcontinent Greater White-fronted Geese

**DOI:** 10.1186/s40462-020-00236-4

**Published:** 2021-01-20

**Authors:** Jay A. VonBank, Mitch D. Weegman, Paul T. Link, Stephanie A. Cunningham, Kevin J. Kraai, Daniel P. Collins, Bart M. Ballard

**Affiliations:** 1 Caesar Kleberg Wildlife Research Institute, Texas A&M University – Kingsville, Kingsville, TX 78363 USA; 2 Present Address: U.S. Geological Survey, Northern Prairie Wildlife Research Center, Jamestown, ND 58401 USA; 3 School of Natural Resources, University of Missouri, Columbia, MO 65211 USA; 4 Louisiana Department of Wildlife and Fisheries, Baton Rouge, LA 70808 USA; 5 Texas Parks and Wildlife Department, Canyon, TX 79015 USA; 6 U.S. Fish and Wildlife Service, Albuquerque, NM 87102 USA

**Keywords:** Movement ecology, Philopatry, Bio-logging, Overall dynamic body acceleration, Accelerometer, Multistate model, Waterfowl

## Abstract

**Background:**

Animal movement patterns are the result of both environmental and physiological effects, and the rates of movement and energy expenditure of given movement strategies are influenced by the physical environment an animal inhabits. Greater white-fronted geese in North America winter in ecologically distinct regions and have undergone a large-scale shift in wintering distribution over the past 20 years. White-fronts continue to winter in historical wintering areas in addition to contemporary areas, but the rates of movement among regions, and energetic consequences of those decisions, are unknown. Additionally, linkages between wintering and breeding regions are generally unknown, and may influence within-winter movement rates.

**Methods:**

We used Global Positioning System and acceleration data from 97 white-fronts during two winters to elucidate movement characteristics, model regional transition probabilities using a multistate model in a Bayesian framework, estimate regional energy expenditure, and determine behavior time-allocation influences on energy expenditure using overall dynamic body acceleration and linear mixed-effects models. We assess the linkages between wintering and breeding regions by evaluating the winter distributions for each breeding region.

**Results:**

White-fronts exhibited greater daily movement early in the winter period, and decreased movements as winter progressed. Transition probabilities were greatest towards contemporary winter regions and away from historical wintering regions. Energy expenditure was up to 55% greater, and white-fronts spent more time feeding and flying, in contemporary wintering regions compared to historical regions. White-fronts subsequently summered across their entire previously known breeding distribution, indicating substantial mixing of individuals of varying breeding provenance during winter.

**Conclusions:**

White-fronts revealed extreme plasticity in their wintering strategy, including high immigration probability to contemporary wintering regions, high emigration from historical wintering regions, and high regional fidelity to western regions, but frequent movements among eastern regions. Given that movements of white-fronts trended toward contemporary wintering regions, we anticipate that a wintering distribution shift eastward will continue. Unexpectedly, greater energy expenditure in contemporary wintering regions revealed variable energetic consequences of choice in wintering region and shifting distribution. Because geese spent more time feeding in contemporary regions than historical regions, increased energy expenditure is likely balanced by increased energy acquisition in contemporary wintering areas.

**Supplementary Information:**

The online version contains supplementary material available at 10.1186/s40462-020-00236-4.

## Background

Deciphering the drivers of animal movements and their consequences on population dynamics is a primary goal of movement ecology, and many conservation and management decisions incorporate movement information into decision-making processes [2]. An individual’s movement pattern is the result of interacting condition-dependent (e.g., landscape structure, seasonality) and phenotypic-dependent (e.g., physiological condition, energetic demands) factors that vary throughout the annual cycle [19, 39, 57]. Two broad movement categories are generally classified in movement analyses that seek to describe movement trajectories: ‘encamped’ and ‘exploratory’ movements [56]. Encamped movements consist of short successive movement distances and high degree turning angles typically exhibited while foraging or resting, whereas exploratory movements consist of longer successive movement distances and low turning angles, indicating directed travel. Decisions to undertake exploratory movements require individuals to predict that conditions (e.g., food resources, predation risk) elsewhere are more favorable than conditions in their present location. Individuals may gather information from conspecifics, landscape cues, or prior experiences to make informed decisions prior to and during exploratory movements [19]. Heterogeneity in conditions at the current area and perceived quality of a future area requires frequent decision-making regarding whether to stay or move throughout environments to maximize fitness (i.e., productivity and/or survival), resulting in movement rates and behavioral time allocation that vary both spatially and temporally [57].

Likewise, energy expenditure varies spatially and is temporally dependent on many factors, including an individual’s physiological state (e.g., egg production or feather molt in birds), movement rates (e.g., migration, disturbance), environmental and landscape conditions (e.g., weather, habitat quality), or season [41, 42, 53]. Individuals may also experience carry-over effects (i.e., lagged effects on fitness of conditions from a previous season in the current or future season; *see* [67]) as a result of performance during previous seasons. For example, in Arctic-nesting geese, energy acquisition and expenditure during the winter period likely affects survival during subsequent spring migration and productivity during the breeding season. Individuals that experience net energy deficits during winter may have insufficient endogenous energy and nutrient stores to be used during migration as well as during incubation and in clutch formation [9]. Carry-over effects that influence fitness at the individual level can scale up to effects at the population level (i.e., cross-seasonal effects), thereby influencing demographics [67]. Although the negative effects of increased energy expenditure can be mitigated through increasing energy acquisition, the rate and quality of energy gained is highly reliant on habitat quantity and quality, which vary spatially and temporally throughout the winter period. Therefore, animals may make movement decisions based on a balance of energetic costs and gains, and the perceived influence of these on fitness. For example, individuals may choose to move among heterogeneous habitats or within the extent of the species’ geographic range if their current location is energetically costly, and movement to a new location is perceived to be beneficial.

In the northern hemisphere, many waterfowl species (ducks, geese and swans) are highly mobile and exhibit seasonal migrations spanning North America [5]. Waterfowl are highly philopatric to natal breeding areas [66], but there is considerable uncertainty regarding the strength of fidelity during the winter period. Robertson and Cooke [65] suggested that geese show a high degree of fidelity to small geographical areas during winter following the ‘local-knowledge’ hypothesis, whereby individuals return to familiar wintering areas to take advantage of previous knowledge of food resources, landscape characteristics (e.g., roost locations), and threats of predation or disturbance [64, 66]. Following this hypothesis, individuals should exhibit high winter fidelity, and would not be expected to make large-scale movements throughout the species’ winter range. Previous studies using band recovery and resight analyses support this hypothesis, and suggest that geese show strong winter site fidelity and make few inter-regional or large-scale movements during winter [3, 36, 68, 73]. Contrary to analyses from band recoveries that are typically limited by infrequent encounters of individuals, studies using tracking devices which allow for increased frequency and duration of location data collection suggest that long-lived waterfowl, particularly geese, may sample several different areas within the species’ winter distribution during the same winter (i.e., low winter fidelity [12, 71]).

Greater white-fronted geese (*Anser albifrons frontalis,* hereafter white-fronts) occur in two populations in North America; the Pacific and Midcontinent Populations. The Pacific Population breeds on the Yukon-Kuskokwim Delta and Bristol Bay Lowlands in Alaska, and migrates along the Pacific coast to winter in the Central Valley of California, and western Mexico. The Midcontinent Population breeds in both taiga and tundra ecosystems, from the interior and north slope of Alaska eastward across the Canadian Arctic, including the Northwest Territories and Nunavut, and migrates down the Central and Mississippi Flyways to wintering areas in the south-central USA [5, 27]. The Midcontinent Population has undergone a large-scale winter distribution shift over the last two decades. White-fronts wintered in the Gulf Coast marshes of Texas and Louisiana before moving inland following agricultural expansion during the 1940s [37]. During the last decade, white-fronts have further shifted their primary wintering range northeastward into the Mississippi Alluvial Valley (MAV), presumably influenced by large-scale landscape modification, predictability of quality food resources (e.g., rice), and hunting pressure. White-fronts have also expanded their winter range into previously uninhabited regions such as the South Texas Brushlands and areas in the Midwest (e.g., Illinois, Indiana). Several coastal and inland regions in Texas and Louisiana still support large subpopulations during winter, but fidelity to and movements among these regions and contemporary wintering regions are largely unknown. Additionally, some question remains as to whether spatial segregation of breeding regions occurs during winter, and its potential impact to population structuring of white-fronts [27]. Understanding wintering distribution with regard to breeding origin is essential for determining population structure, gene flow among breeding regions, and their influence on demographics [36, 65, 73].

Understanding inter-regional movement is important for future conservation and management of white-fronts, particularly with regard to a continued distribution shift during winter. Additionally, because land use practices and human-induced disturbances vary dramatically among wintering regions, there may be differential energetic costs to white-fronts among wintering regions. In this paper, we describe the winter movements of white-fronts using location data collected from state-of-the-art tracking devices. Our objectives were to 1) determine if daily movement distances of white-fronts varied throughout the winter period, 2) determine the probability of movements among ecologically distinct wintering regions, 3) compare energy expenditure among wintering regions and determine how differences in behaviors among regions translate to differences in energy expenditure, and 4) evaluate the linkages between wintering and breeding areas. We predicted that daily movement distances would increase as winter progressed (e.g., as a function of food depletion requiring increased movement to locate food resources [22, 34]), and decrease prior to spring migration as individuals begin refueling energy stores to prepare to migrate [18, 58]. We also predicted that individuals would have higher probability of within-season movements to contemporary wintering regions (e.g., MAV, Chenier Plain), as opposed to historical regions (e.g., Lower Texas Coast, Texas Mid-coast). Also, because we predict considerable movement among regions, white-fronts should exhibit admixture of breeding populations where breeding affiliations are not spatially segregated among wintering regions [74]. Additionally, we hypothesized that energy expenditure would be lower in the contemporary wintering regions than in historic wintering regions, reflective of an energetic benefit to the winter distribution shift.

## Methods

### Goose capture and tracking device specifications

**Fig. 1:**
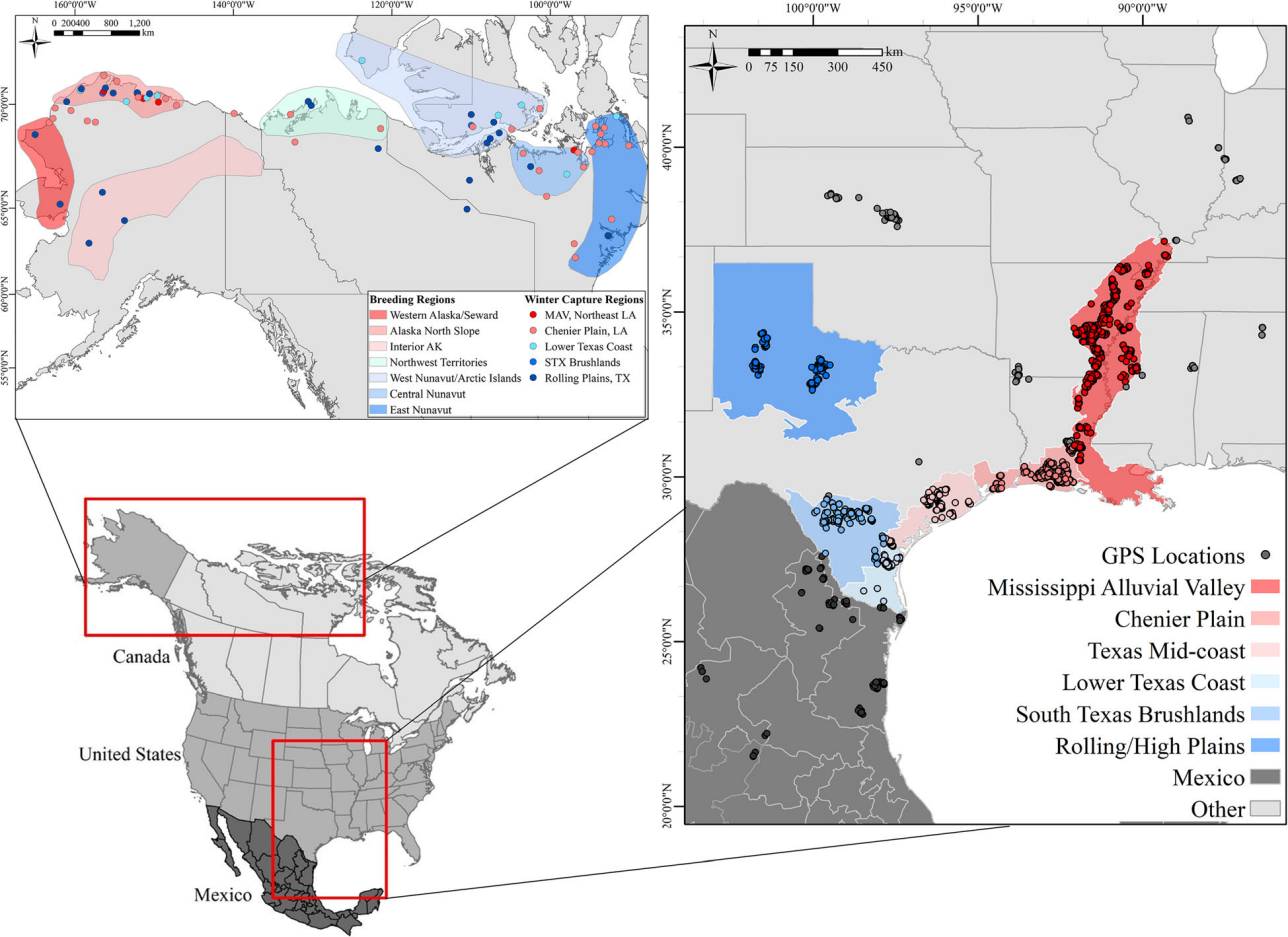
Study area of greater white-fronted goose (*Anser albifrons frontalis*) wintering regions (right) in the southern United States and Mexico with GPS locations (one GPS location per goose per day) colored by region, and breeding areas across Alaska and Canada (top) with one representative GPS location per goose of white-fronts during summers 2017–2019

We captured 97 after-hatch-year white-fronts (79 females, 18 males) in three regions of Texas (Rolling Plains, Lower Texas Coast, and South Texas Brushlands) and one region of Louisiana (Chenier Plain) from October to February 2016–2018 using rocket nets and modified leg snares (Fig. 1). Tracking devices were 36–54 g solar powered Global Positioning System-Acceleration-Global System for Mobile communication (GPS-ACC-GSM) units that were integrated into a neck collar design (Cellular Tracking Technologies, Rio Grande, New Jersey, USA, and Ornitela OrniTrack-N38, Vilnius, Lithuania). We fit tracking devices to a single sex (although females were our priority) during each unique capture event to ensure independence among tagged individuals, because white-fronts maintain family associations throughout winter and have long-term pair bonds [10]. When multiple geese were captured at the same time, we released all captured geese (i.e., juveniles, adult males and females) in unison to retain family group and pair bond structure. Locations were recorded at 30-min intervals (i.e., 48 locations/day), or 1 h intervals (i.e., 24 locations/day) at ±7.2 and 6.5 m accuracy for CTT and Ornitela devices, respectively [69]. Tri-axial ACC data were collected at 6 min intervals for a 3 s duration at 10 Hz (240 fixes/day) in G-force (CTT devices) or millivolts (Ornitela), and we used brand- and tag-specific calibrations to transform both device types to m/s^2^ [70]. We censored GPS and ACC data from the time of release until normal activity resumed on an individual basis while white-fronts acclimated to wearing devices, which ranged from 1 to 7 days. Following device attachment, geese typically traveled from the capture site to a nearby wetland, remaining there without making normal daily foraging flights, presumably until they became acclimated to the device at which point they resumed normal movements between roosting wetlands and agricultural fields used for foraging. Therefore, we define normal activity as conducting at least one flight between roosting and foraging locations following device attachment. We defined the start of the winter period following a southward migratory movement from staging areas in prairie Canada, and ≥ 4 days without additional large-scale movements (i.e., > 50 km, [26]) southward at ≤40°0′0″ N, from the time of device deployment (excluding device acclimation period) until geese migrated northward outside of wintering regions, or until 28 February if geese remained in wintering areas.

### Determination of winter and breeding regions

Wintering regions included the MAV of Arkansas, Louisiana, and Mississippi, Chenier Plain of Louisiana and Texas, Texas Mid-coast, Lower Texas Coast, Rolling/High Plains of Texas, South Texas Brushlands, and Mexico; any GPS locations collected outside of these regions were classified as Other (Fig. 1). We considered these as independent regions because they are ecologically distinct, support different agricultural crops and wetland types (e.g., rice and agricultural wetlands in the MAV, peanuts and playa wetlands in the Rolling/High Plains), and have been delineated as distinct regions for management and conservation purposes (e.g., Federal flyway systems, bird conservation regions, Joint Venture regions). We used shapefiles of region extent from the Gulf Coast Joint Venture for the Lower Texas Coast, Texas Mid-Coast, and Chenier Plain, and used the United States Environmental Protection Agency Ecoregion IV shapefiles for the South Texas Brushlands, Rolling/High Plains of Texas, and the MAV, and the international border for Mexico from ArcMap 10.3.1 (ESRI, Redlands, California).

We determined the breeding region of all white-fronts based on GPS data in order to link with winter distribution. Due to extremely limited GSM coverage throughout the white-front breeding range, only white-fronts with functional devices that survived through the summer breeding season and successfully migrated southward to GSM coverage were included because we could not detect individuals that experienced transmitter failure or mortality during the breeding season. In total, 39 of 97 white-fronts used in movement analyses during winter herein provided GPS data during summer to determine their summer breeding region. Tracking and captures continued in winter 2018–2019, and we included 36 additional white-fronts, including white-fronts captured in the MAV of northeast Louisiana, to strengthen analysis of breeding regions, resulting in 75 total individuals (i.e., these additional 36 individuals were not used in winter analyses). We classified individuals into one of seven breeding regions modified from [27]; Fig. 1) and calculated the proportion of individuals captured from each wintering region that associated with each breeding region. We then calculated the proportion of GPS locations within each winter region from the total GPS locations of geese assigned to each breeding region to help evaluate the affiliations between breeding and wintering areas.

### Daily movement distances

For consistency, we resampled individuals with more frequent GPS location collection schemes to 1-h intervals and removed obvious GPS outliers due to transmitter error, totaling 130,599 GPS locations across two winters (see Additional File Fig. 1). The mean number of locations per individual was 1346 and ranged 18–8155 locations. We calculated successive step lengths (km) for each individual separately for both winters, removed successive step length outliers due to missing GPS fixes (all outliers were ≥ 4 h gaps), then calculated the total distance traveled per individual per day, and calculated the mean across all individuals per day using the R packages adehabitatLT and move [15, 47] in Program R and RStudio interface (v. 3.5.2; R Core Team [63]). The mean total daily distance traveled was log-transformed to meet the assumption that residuals were approximately normal, tested using a Shapiro-Wilks normality test, and total daily movement distances were assumed to be independent for each day. We developed a winter date index, which began on the earliest date of tracking device deployment (excluding the acclimation period; day 1) and ended on 28 February each winter (day 137). We further censored dates when ≤3 individuals provided data to reduce high variability in total daily distance due to low sample sizes at the beginning of winter when transmitters were progressively deployed, and at the end of winter when individuals began departing northward ending their winter period. To determine if the amount of movement by white-fronts changed throughout winter, we used separate simple linear regressions for each winter to explain mean total daily distance moved as a function of the winter date index. We used a backward elimination of polynomial terms beginning with a cubic term in the linear regression model to examine the relationship of daily movement distances and winter date index in each year [38, 45], and evaluated models based on *p*-value significance. Additionally, for each winter, we calculated the proportion of individuals that made at least one inter-regional movement.

### Multistate capture-recapture model

We developed daily capture histories for each individual by subsetting GPS locations to one location per day that was closest to midnight to meet the assumption that an individual must survive before transitioning to a state in the next time period. To determine transition probabilities among wintering regions, *ψ*, we developed a Bayesian multistate capture–recapture model with nine states in JAGS (ver. 4.2.0 [62]) using the jagsUI package [44]. We assigned capture histories according to states: “1”– observed in South Texas Brushlands, “2”– observed in the MAV, “3”– observed in the Chenier Plain, “4”– observed in the Texas Mid-coast, “5” – observed in Other areas, “6” – observed in Mexico, “7” – observed in the Lower Texas Coast, “8” – observed in the Rolling/High Plains, and “9” – not observed. Only one state was assigned per day. We then scaled daily capture histories into weekly (7 day) capture histories (i.e., one state per week of winter) and assigned a single state to each week. If a transition occurred within a week, the capture history reflected the transitioned-to state even if the individual returned to the original state during the same week. For example, the capture history ‘BBBBAAA’ received state A for the week, while the capture history ‘AAAABBA’ received state B for the week, to capture the transition information. If more than two state transitions occurred during a week, the final transition was recorded for the week. For example, ‘AABBCCC’ received state C, as the B state transition was considered an intermediate step to state C. We developed capture histories specific to each year, although some individuals contributed ~ 2 years of data. Thus, while we deployed devices on geese in four regions (states), some individuals could start their capture history in other regions (i.e., beginning the second winter). Furthermore, while not all possible combinations of transitions were observed in our data, we did not restrict the analysis to only estimate observed transition probabilities because all transitions were biologically possible [72]. We developed our nine-state model using the following state and observation equations,

1 $$ {z}_{i,{f}_i}={f}_{s_i} $$

2 $$ {z}_{i,t+1}\mid {z}_{i,t}\sim \mathrm{categorical}\left({\Omega}_{z_{i,t},1\dots S,i,t}\right), $$

where *z*_*i*, *t*_ was the true state of individual *i* at time *t*, ${f}_{s_i}$ was the observed state at the first encounter of individual i, *S* was the number of true states (i.e., *S* = 9), and Ω was the four-dimensional state-transition matrix comprising the starting and ending states, individual *i* and time *t* (Table 1 in Additional Files, [46]). We linked the true state with the observed state via the observation equation,

**Table 1:** Winter capture regions and subsequent summer breeding regions of 75 greater white-fronted geese (*Anser albifrons frontalis*) captured and fit with GPS-ACC-GSM transmitters during winters 2016–2019. Values and percentages indicate the number and percent of individuals breeding in each specific region in relation to the total number of individuals captured in that region (*n*). Regions include Interior Alaska, USA (Interior AK), Western Alaskan coast and Seward Peninsula, Alaska, USA (West AK/Seward Pen.), the North Slope of Alaska, USA (AK North Slope), mainland Northwest Territories, Canada (NWT), Western Nunavut and high Arctic islands of Nunavut, Canada (West NU/Arctic Isl), central Nunavut, Canada, and eastern Nunavut, Canada

		Breeding Region						
Capture Region	*n*	Interior AK	West AK/ Seward Pen.	AK North Slope	NWT	West NU/ Arctic Isl.	Central NU	East NU
Rolling/High Plains, TX	23	3 (13.0%)	2 (8.7%)	7 (30.4%)	2 (8.7%)	5 (21.7%)	3 (13.0%)	1 (4.3%)
Chenier Plain, LA	35	0 (0.0%)	2 (5.7%)	10 (28.6%)	2 (5.7%)	5 (14.3%)	8 (22.9%)	8 (22.9%)
Lower Texas Coast, TX	10	0 (0.0%)	0 (0.0%)	5 (50.0%)	0 (0.0%)	3 (30.0%)	1 (10.0%)	1 (10.0%)
MAV^a^, Northeast LA	5	0 (0.0%)	0 (0.0%)	4 (80.0%)	0 (0.0%)	0 (0.0%)	0 (0.0%)	1 (20.0%)
South Brushlands, TX	2	0 (0.0%)	0 (0.0%)	1 (50.0%)	0 (0.0%)	1 (50.0%)	0 (0.0%)	0 (0.0%)
**Total**	75	3 (4.0%)	4 (5.3%)	27 (36.0%)	4 (5.3%)	14 (18.7%)	12 (16.0%)	11 (14.7%)

^a^Mississippi Alluvial Valley

3 $$ {y}_{i,t}\mid {z}_{i,t}\sim \mathrm{categorical}\left({\Theta}_{z_{i,t},1\dots O,i,t}\right), $$

where *y*_*i*, *t*_ was the observed state of individual *i* at time *t*, Θ was the four dimensional observation matrix (i.e., the true state, the observed state, *i* and *t*), and *O* was the number of observed states (i.e., *O* = 8; Table 2 in Additional Files, [46]). We estimated transition probabilities using vague normal priors and a multinomial logit link function constrained so that the sum of all transition probabilities was < 1 [46].

**Table 2:** Wintering regions and subsequent summer breeding regions of 39 greater white-fronted geese (*Anser albifrons frontalis*) captured and fit with GPS-ACC-GSM transmitters during winters 2016–2018. Values are percentages of the total number of GPS locations in each winter region (GPS) that correspond to breeding regions from the number of geese that wintered there (*n*). Regions include Interior Alaska, USA, Western Alaskan coast and Seward Peninsula, Alaska, USA (West AK/Seward Pen.), the North Slope of Alaska, USA, mainland Northwest Territories, CA (NWT), Western Nunavut and high Arctic islands of Nunavut, CA (West NU/Arctic Isl.), central Nunavut, CA, and eastern Nunavut, CA

			Winter Region							
Breeding Region	*n*^a^	GPS^b^	MAV	Chenier Plain	Texas Mid-coast	Lower Texas Coast	South Texas Brushlands	Rolling/High Plains	Mexico	Other
Interior Alaska	3	6122	26.2	0.0	0.5	0.03	0.3	32.9	39.7	0.3
North Slope Alaska	15	27,000	10.1	19.0	9.8	9.2	33.5	13.0	0.0	5.4
NWT	3	1735	24.8	6.9	0.0	0.0	0.0	68.2	0.0	0.06
West NU/Arctic Isl.	8	24,805	15.3	24.4	1.7	21.1	15.8	19.4	0.02	2.4
Central Nunavut	6	19,259	19.3	9.4	0.04	0.3	24.0	11.2	0.0	35.7
East Nunavut	4	3696	1.0	80.5	5.4	10.1	0.0	0.0	0.0	3.1
West AK/Seward Pen.	0	–	–	–	–	–	–	–	–	–

^a^ Number of geese in each breeding region that provided winter GPS data included in analyses

^b^ Number of GPS locations during winter from geese (*n*) associated with each respective breeding region

We estimated state-specific survival and resighting probabilities in the multistate model using uniformly distributed vague priors (mean 0, standard deviation 1 [46];). Because we could not decipher transmitter failure from true mortality, we do not provide any interpretation for survival or resighting probabilities. However, survival and resighting probabilities are necessary to calculate transition probabilities. We used three Markov chain Monte Carlo chains with 450,000 iterations, 9000 burn-in iterations, and a thinning interval of 10 to derive posterior summaries. We confirmed convergence of chains using the Gelman–Rubin statistic with $\hat{R}$ ≤ 1.10 [13], and by assessing trace plots. We present mean transition probabilities with associated 95% credible intervals.

### Energy expenditure

Overall dynamic body acceleration (ODBA) is a proxy for energetic expenditure, which is highly correlated with the rate of oxygen consumption, and therefore, metabolic rate [33, 75]. We used a subset of 56 white-fronts (*n* = 37 in 2016–2017; *n* = 19 in 2017–2018; 52 after-hatch-year females, 4 after-hatch-year males) with temporally-matched GPS and ACC data to determine energy expenditure per region (i.e., some devices that provided GPS data did not provide ACC data [69]). Ornitela devices measured ACC values bounded by maximum and minimum values (-2048 and 2048 mV, respectively), while CTT device measurements were unbounded and therefore unbiased to behaviors inducing large ACC measurement peaks (e.g., flying). To correct the Ornitela ACC measurements to an unbounded distribution, we used quantile mapping in the qmap package [31] using smoothing splines as the transformation function to transform the distribution of bounded Ornitela ACC values to the unbounded CTT distribution, and visually assessed cumulative density function plots to assess transformation fit [21, 31, 59]. After transformation, we combined CTT and Ornitela datasets. We calculated ODBA using the formula:

4 $$ ODBA=\left| DAx\right|+\left| DAy\right|+\mid DAz\mid $$

where DA is dynamic acceleration for each axis x, y, z, after subtracting static acceleration due to gravity from each raw ACC measurement, with a moving average calculated from 1 s of ACC values for each axis. We used location information to determine the region each individual was present in each day similar to the multistate model, and calculated mean ODBA from all ACC bursts per individual per region per day (i.e., daily ODBA). Not all individuals were present each day of winter depending on transmitter deployment date and data quality, and individual-specific data in relation to our study period can be found in Additional File 1. We used a linear mixed-effects model in the lme4 package [6] with individual ID and winter (two levels) as crossed-random effects to model the effect of region on daily ODBA, with the region MAV set as the reference level so that comparisons could be made to the primary contemporary wintering region, and set α = 0.05. We centered, standardized, and used an inverse hyperbolic sine transformation to account for heavy-tailed model residuals, which then met normality assumptions [60]. We compared means among all regions using the multcomp package [40].

To determine the influence of behaviors on energy expenditure by region, we used video-recorded behaviors from captive and wild white-fronts to pair ACC signatures with known behaviors, and used random forest classification algorithms with > 95% accuracy to predict behaviors of unclassified ACC data (see [21, 69] for detailed methods). Briefly, we collected 119 h of video footage and classified behaviors from two captive individuals and 18 wild individuals in weather conditions suitable for video recording during winter 2017–2018. We then assigned one of four specific behaviors (i.e., foraging, walking, stationary, and flight) to white-fronted geese continuously in video footage. All assigned behaviors were temporally matched to ACC bursts using the program JWatcher [11] and video timestamps [69]. We then used these known behavioral signatures to classify ACC bursts from wild, tagged geese to observed behaviors via random forest classification [49]. Finally, we; compared ODBA derived from energetically costly behaviors, flight and foraging, among regions. We then calculated the mean daily proportion of time spent foraging and flying per region, and separately regressed those proportions on the difference in back-transformed beta estimates of ODBA from the MAV (reference category in the linear mixed-effects model), using beta regression with a logit link [20] in the betareg package [78] to determine the influence of foraging and flying on the variation in ODBA among regions.

## Results

### Daily movement

**Fig. 2:**
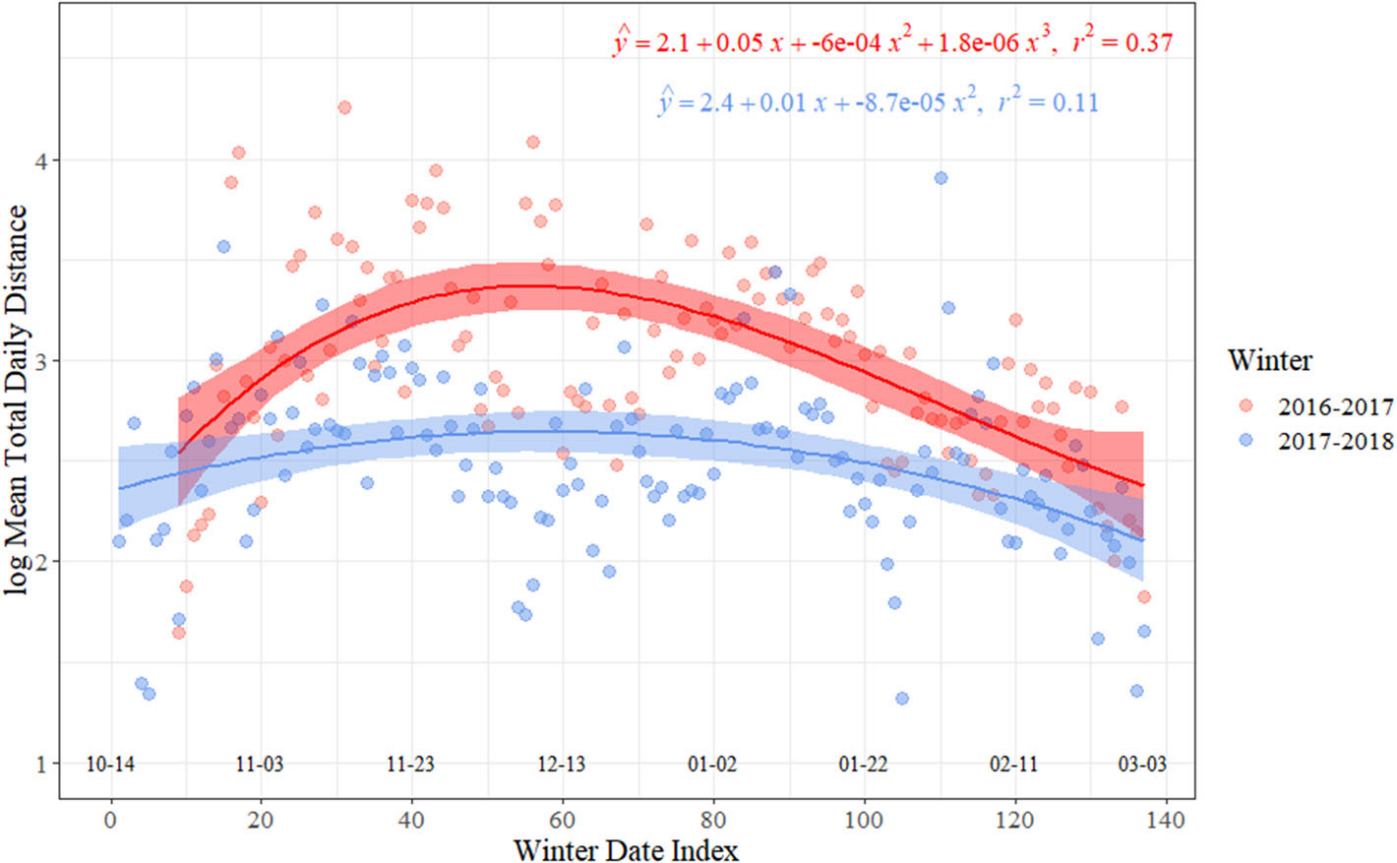
Mean total daily movement (km on log scale) and 95% confidence interval as a function of winter date index for greater white-fronted geese (*Anser albifrons frontalis*) for winter 2016–2017 (red) and winter 2017–2018 (blue). Dates along the x-axis correspond to winter date index

During winter 2016–2017, daily movements exhibited a significant cubic relationship with winter date index (R^2^ = 0.37, *p*-value = < 0.001, F_3,125_ = 24.52), where movement increased during early winter, with peak predicted movement occurring on 01 Dec 2016, followed by a decrease throughout winter, with the rate of decrease lessening immediately prior to the end of winter (Fig. 2). During winter 2017–2018, daily movements exhibited a significant quadratic relationship, but explained less variation (R^2^ = 0.11, *p*-value < 0.001, F_2,134_ = 8.24), where movement slightly increased during early winter, with peak predicted movement occurring on 11 Dec 2017, followed by a slight decrease throughout the remainder of winter (Fig. 2).

### Region-specific transition probabilities

During winter 2016–2017, 32.3% (*n* = 20 of 62) of tagged white-fronts made at least one inter-regional movement, but the percentage was higher during winter 2017–2018, when 50.9% (*n* = 28 of 55) of white-fronts moved among regions. Across both winters, 38.9% (*n* = 7 of 18) of males and 49.4% (*n* = 39 of 79) of females made at least one movement among regions. Of the white-fronts that made regional movements, individuals used an average of 2.8 ± SE 0.02 regions and a maximum of 6 regions. The number of individuals that wintered in each region, including individuals that visited multiple regions per winter, varied whereby the Chenier Plain (*n* = 48) contained the most individuals, followed by the MAV (*n* = 30), Lower Texas Coast (*n* = 29), Other (*n* = 19), South Texas Brushlands (*n* = 16), Rolling/High Plains (*n* = 16), Texas Mid-coast (*n* = 12), and Mexico (*n* = 8).

**Fig. 3:**
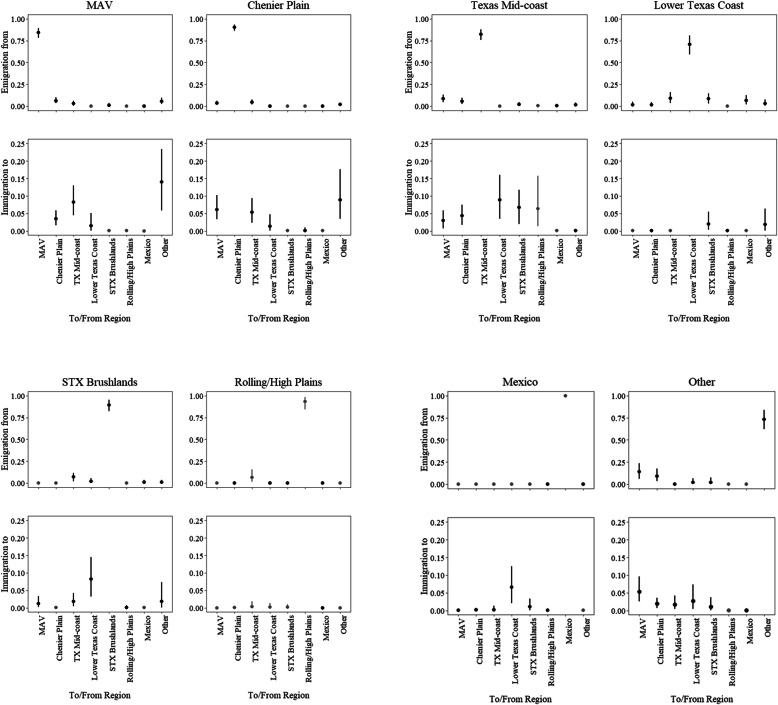
Mean posterior estimates (and 95% credible intervals) of weekly transition probabilities for greater white-fronted geese (*Anser albifrons frontalis*) among eight ecologically distinct wintering regions during winters 2016–2018. Estimates are grouped by immigration to and emigration from each region. Emigration from a region to the same region indicates weekly fidelity, or the probability of staying in that region. Gray colored point estimates and confidence intervals indicate parameters which did not fully converge to the posterior distribution. Note y-axis scales vary between immigration and emigration plots. MAV = Mississippi Alluvial Valley and STX Brushlands = South Texas Brushlands

Convergence diagnostics were satisfactory for 42 of 64 total *ψ* estimates ($\hat{R}$ ≤ 1.10), however 22 estimates of *ψ* did not fully converge ($\hat{R}$ = 1.15–1.71). Of the transitions that did not converge, 19 transitions returned the prior (*ψ ≤* 0.001) because those transitions did not occur in our data and were considered *ψ* = 0.00. We did not interpret these transitions. Weekly regional fidelity (mean *ψ*, 95% credible interval) was greatest in Mexico (0.99, 0.99–1.00), Rolling/High Plains (0.93, 0.84–0.98), and Chenier Plain (0.90, 0.86–0.93), intermediate for the South Texas Brushlands (0.89, 0.82–0.95), MAV (0.84, 0.78–0.89) and the Texas Mid-coast (0.82, 0.76–0.88), and lowest for Other (0.73, 0.62–0.84) and the Lower Texas Coast (0.70, 0.59–0.81; Fig. 3).

The cumulative probability of white-fronts immigrating to a region from all other regions was greatest for the Texas Mid-coast (0.292), MAV (0.273), Chenier Plain (0.216), moderate for South Texas Brushlands (0.132), Other (0.128), Mexico (0.081), and lowest for Lower Texas Coast (0.037), and Rolling/High Plains (0.007). The cumulative probability of emigration from a region to any other region was greatest for the Lower Texas Coast (0.293), Other (0.266), Texas Mid-coast (0.177), moderate for MAV (0.156), South Texas Brushlands (0.109), and Chenier Plain (0.099), and lowest for Rolling/High Plains (0.066) and Mexico (0.001). The MAV, Chenier Plain, and Other regions all had relatively large probabilities of movement to each other (0.03–0.14; Fig. 3). The probability of immigrating to the MAV was greatest from Other (0.14, 0.06–0.23) and the Texas Mid-coast (0.08, 0.04–0.13; Fig. 3). Immigration to the Texas Mid-coast was variable but similar among regions, receiving individuals from nearly all regions, including the Lower Texas Coast (0.09, 0.03–0.16), South Texas Brushlands (0.07, 0.02–0.12), Chenier Plain (0.04, 0.02–0.08), and MAV (0.03, 0.01–0.04; Fig. 3). Emigration from the Texas Mid-coast was greatest to the MAV (0.08, 0.04–0.13) and Chenier Plain (0.05, 0.02–0.09; Fig. 3). Individuals emigrating from the Lower Texas Coast primarily went to the Texas Mid-coast (0.09, 0.03–0.16), the South Texas Brushlands (0.08, 0.03–0.15) and Mexico (0.07, 0.02–0.13), and received little immigration (Fig. 3). In the Rolling/High Plains, we detected no immigration from other wintering regions throughout the study, and individuals emigrating from there had the greatest probability of transitioning to the Texas Mid-coast; however, this estimate did not fully converge. Similarly, no individuals emigrated from Mexico to any other region, and immigration probabilities into Mexico were greatest from regions in closest proximity (i.e., the Lower Texas Coast [0.07, 0.02–0.13] and South Texas Brushlands [0.02, 0.00–0.03]; Fig. 3).

### Energy expenditure and behavior

**Fig. 4:**
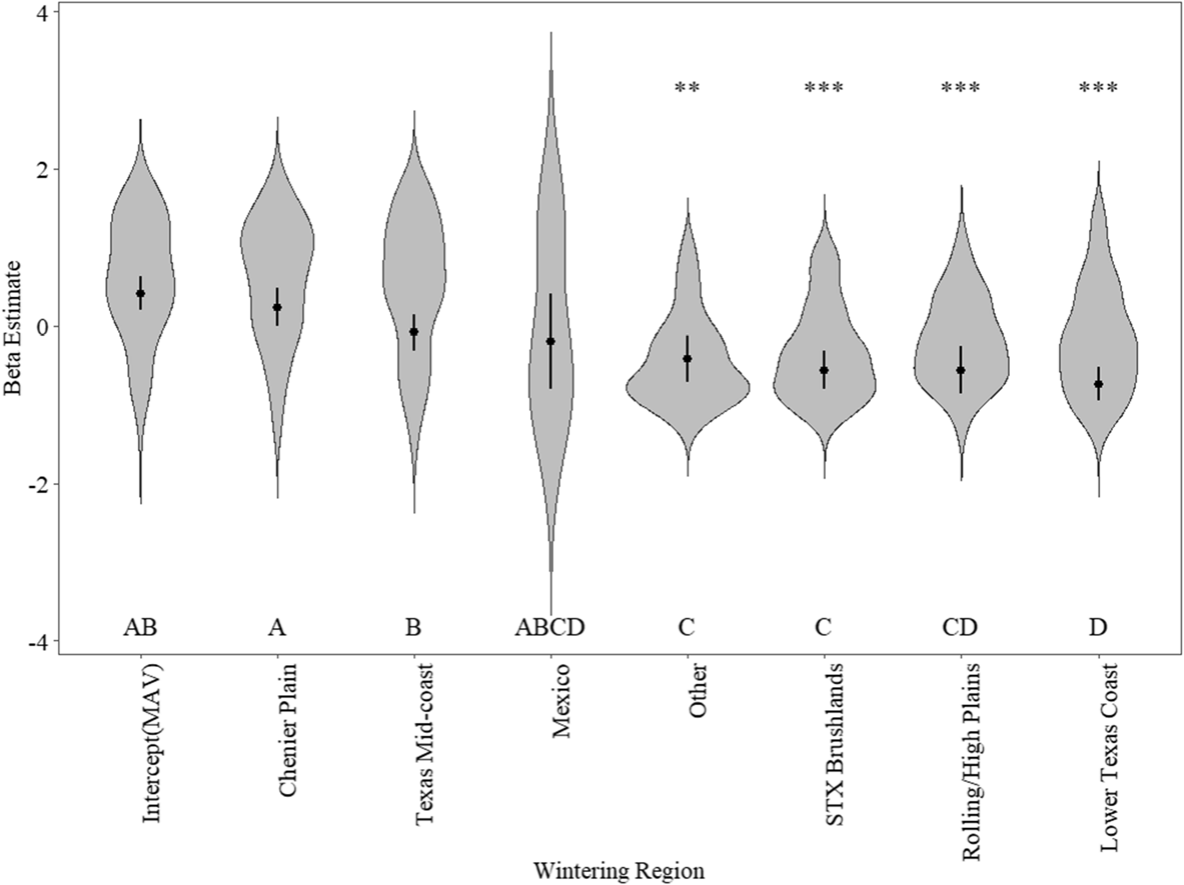
Beta estimates and 95% confidence intervals from linear mixed-effects modelling and kernel density distribution of daily overall dynamic body acceleration (ODBA) from individual greater white-fronted geese (*Anser albifrons frontalis*) in eight wintering regions during winters 2016–2018. Asterisks indicate significantly different (α = 0.05, ** = *p*-value ≤0.01, *** = *p*-value ≤0.001) estimates from the intercept, and letters indicate pairwise comparisons where different letters are significantly different from one another. MAV = Mississippi Alluvial Valley, STX Brushlands = South Texas Brushlands

We analyzed ODBA from 1622 goose days among all 8 regions and found that it varied significantly among regions (*p*-value < 0.001, F_7, 329_ = 22.69). Post-hoc pairwise comparisons indicated two general levels of ODBA among regions (Fig. 4). Regions where ODBA was relatively high included Chenier Plain and Texas Mid-coast, which had values similar to the reference region (MAV). Estimates of ODBA were 33% lower in Other, 42% lower in the South Texas Brushlands and Rolling/High Plains, and 55% lower in the Lower Texas Coast than in the MAV. Mexico had high variation in ODBA due to a small sample size of ACC data and, as a result, was similar to all other regions. Region explained 17.0% (marginal R^2^) and 34.6% (conditional R^2^) of the variation in ODBA, indicating that there are additional individual and between-winter variations in energy expenditure.

**Fig. 5:**
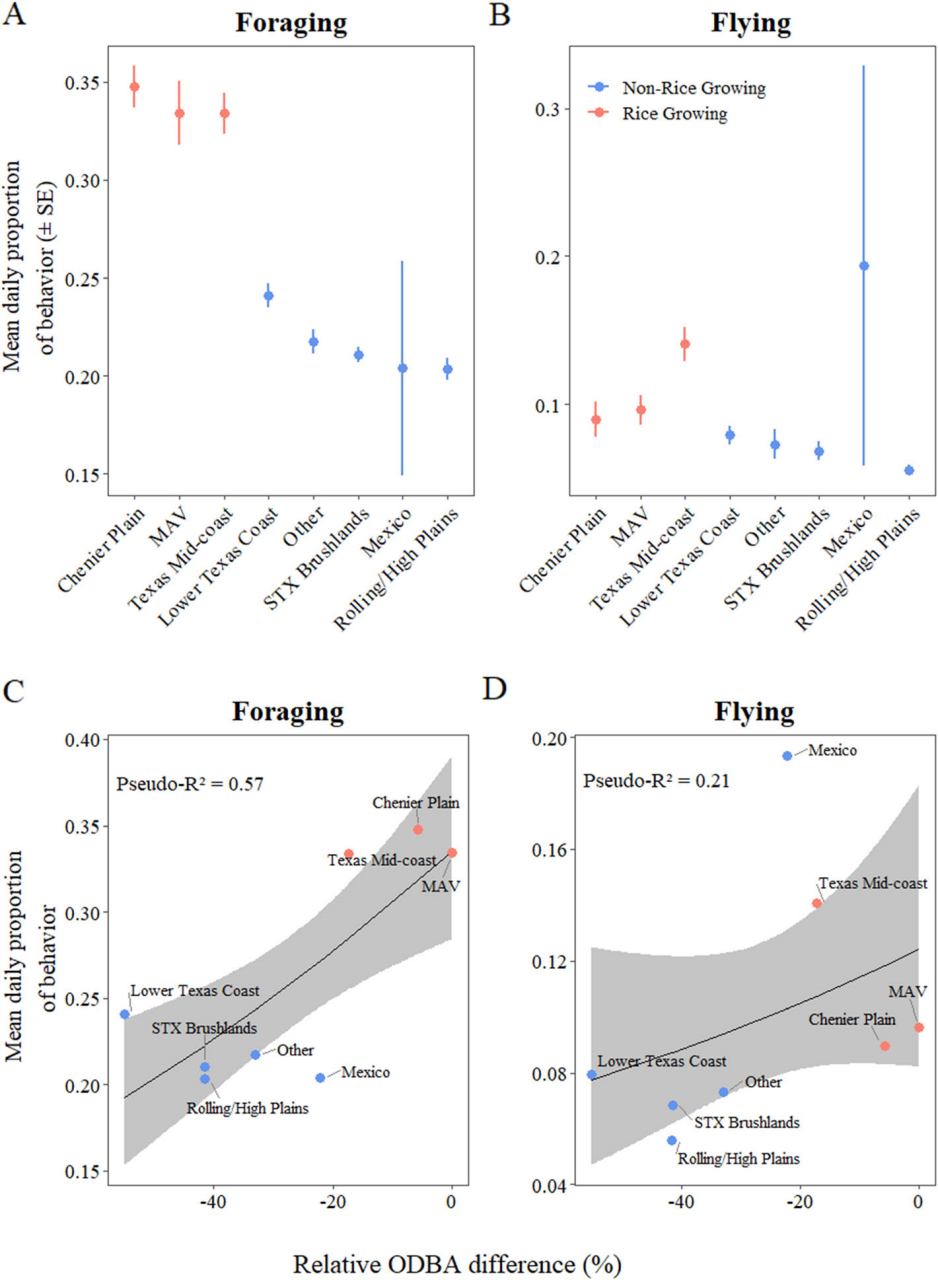
Mean daily proportion (± SE) of time spent foraging (A) and flying (B) in each wintering region, and the relationship via beta regression (± 95% confidence interval) between time spent foraging (C) and flying (D) with the difference in daily energy expenditure from the Mississippi Alluvial Valley (MAV), grouped by whether the region produces rice agriculture or not

Depending on region, white-fronts spent an average of 20.4–34.8% of the day foraging (Fig. 5a), and 4.3–7.0% of the day in flight during winter (Fig. 5b). White-fronts spent the most time foraging in the Chenier Plain (mean 34.8 ± SE 1.1%), the MAV (33.4 ± 1.6%) and the Texas Mid-coast (33.4 ± 1.0%), followed by the Lower Texas Coast (24.1 ± 0.6%), Other (21.7 ± 0.6%), South Texas Brushlands (21.1 ± 0.4%), Rolling/High Plains (20.4 ± 0.6%) and Mexico (20.4 ± 5.5%; Fig. 5). White-fronts spent the most time flying in Mexico (19.3 ± 13.5%), the Texas Mid-coast (14.1 ± 1.1%) and the MAV (9.6 ± 0.9%), followed by the Chenier Plain (9.0 ± 1.1%), Lower Texas Coast (7.9 ± 0.6%), Other (7.3 ± 1.0%), South Texas Brushlands (6.8 ± 0.6%), and Rolling/High Plains (5.6 ± 0.4%; Fig. 5). On average, white-fronts spent 52% more time foraging and 57% more time flying in rice growing regions than in all other regions (Fig. 5a and b). Mean daily proportion of time spent feeding explained significant variation in the difference in mean daily ODBA between region and the MAV, and this relationship was positive (Pseudo-R^2^ = 0.57, *p*-value < 0.001, Z = 3.427, Fig. 5). We found little evidence that mean daily proportion of time spent flying explained variation in the difference in mean daily ODBA between region and the MAV (Pseudo-R^2^ = 0.21, *p*-value = 0.214, Z = 1.243, Fig. 5).

### Capture and breeding regions

We determined seven regions where white-fronts spent the subsequent summer breeding season after winter capture, which spanned the entire breeding range of white-fronts (Fig. 1). Individuals in all capture regions did not exhibit explicit segregation among breeding regions (Table 1). White-fronts from particular breeding regions spent time in many winter regions, while others were more limited (Table 2), revealing a high degree of admixture of breeding affiliations among most winter regions. For example, white-fronts breeding in Eastern Nunavut (*n* = 4) spent 80.5% of winter in the Chenier Plain, while white-fronts from Western Nunavut/Arctic Islands (*n* = 8) spent 24.4% of winter in the Chenier Plain, 21.1% on the Lower Texas Coast, 19.4% in the Rolling/High Plains, 15.8% in the South Texas Brushlands, and 15.3% in the MAV (Table 2). Individuals breeding in Interior Alaska (*n* = 3) were only captured in the Rolling/High Plains; however, the Rolling/High Plains was not the predominant wintering region of geese that summered in Interior Alaska, as those white-fronts spent 39.7% of winter in Mexico, 32.9% in Rolling/High Plains, and 26.2% in the MAV (Table 2). Over 70% of all white-fronts captured summered in just three breeding regions; the Alaskan North Slope (36.0%), Western Nunavut/Arctic Islands (18.7%), and Central Nunavut (16.0%), with fewer white-fronts in other areas (Table 2). Ten individuals provided breeding information for two back-to-back summer periods, and all 10 returned to the same breeding region as the prior summer.

## Discussion

Using rich GPS and acceleration data, we found that individuals in the North American Midcontinent Population of greater white-fronted geese frequently make landscape-level movements throughout their entire winter range and among ecologically-distinct wintering regions. The general pattern of movement was eastward from regions in Texas to the Mississippi Alluvial Valley, yet the latter region had the greatest energy expenditure. White-fronts exhibited considerable plasticity in their wintering strategies among individuals, from frequent regional movements to high within-season regional fidelity. We suggest that individual white-fronts generally operate under a ‘landscape-knowledge’ approach, whereby they have ‘local-knowledge’ of many areas throughout their range, and make movements between regions determined by resource conditions, weather, disturbance, predation levels, individual heterogeneity, or by the presumed balance of energetic costs and benefits of moving throughout winter [8, 29, 35]. Thus, our results suggest that white-fronts are capable of sampling large geographic areas to make decisions regarding their wintering locations on a within- and among-winter basis.

Consistent with our hypothesis, we found that immigration rates were relatively high for contemporary wintering regions compared to historical wintering areas, with the exception of the Texas Mid-coast, which had the highest cumulative immigration probability. While the Texas Mid-coast is a historical wintering area, it had consistent immigration and emigration rates from nearly all other wintering regions, and given its central location relative to all wintering regions, it may serve as a central hub for individuals transitioning among regions. In general, regions that had relatively high immigration rates also had relatively high emigration rates. Further, immigration and emigration tended to be reciprocal between specific wintering regions, with interchange occurring with > 3 regions in most cases. The largest emigration probabilities were derived from the Lower Texas Coast, Other, and Texas Mid-coast. With the exception of the Texas Mid-coast, the largest immigration probabilities were to, and between, the MAV and Chenier Plain, consistent with the observed winter distribution shift to the MAV and away from historical winter regions. White-fronts exhibited greater within-season fidelity to western regions, including Mexico, Rolling/High Plains, and the South Texas Brushlands. Greater fidelity to some regions may be a function of resource predictability and landscape composition as has been observed in other waterfowl, shorebird, and passerine species [23, 24, 77]. Transition probability estimates should be considered minimum estimates that could be larger than presented here, because we captured individuals in different regions throughout the winter period and therefore cannot infer whether an individual began winter in that region, or had completed movements among regions prior to capture.

Several studies have used multistate capture-recapture models to investigate transition probabilities between specific geographic areas in birds [4, 30, 50] and more specifically geese [36, 73]. However, these studies investigated movements among regions from year to year, which is an artifact of using band-recovery or resight data across large scales. Williams et al. [73] investigated winter site fidelity in lesser snow geese that nested on Wrangel Island, Russia, and determined that high annual winter site fidelity (≥ 97% to all regions) from analysis of band recovery data could not explain an observed northward distribution shift from California wintering areas to the Skagit-Fraser region of British Columbia, Canada and Washington, USA. We estimated fidelity to winter regions at the weekly scale instead of the annual scale, and although our estimates of within-season fidelity were similarly high for some regions, several regions exhibited much lower weekly fidelity compared to annual fidelity. This suggests that interpreting winter fidelity at coarser temporal scales may be misleading, as individuals make fine-scale decisions (i.e., at least weekly) regarding whether or not to move among regions. Using GPS tracking devices instead of marker recovery/resight information allows researchers to know the locations of individuals in near real-time with high precision, without relying on many unpredictable factors influencing marker reporting, and provides high temporal and spatial resolution of movements.

Contrary to our hypothesis, energy expenditure was significantly greater in contemporary wintering regions than in most other historical wintering regions. Despite higher immigration probability to the MAV than many other regions, we found that energy expenditure was 5.7–55.1% greater in the MAV than other regions, and was followed closely by the Chenier Plain, and Texas Mid-coast. Therefore, greater ODBA in specific wintering regions can be proximately attributed to varying time activity budgets, which could be explained by regional habitat quantity and quality, disturbance, or environmental conditions. Optimal foraging theory and energy landscape theory predict that animals should forage on foods that maximize energy intake per unit cost to acquire, and in areas of the energy landscape that result in energetic profitability, where gains outweigh costs [51, 54, 76]. During the same period as this study, Massey et al. [55] showed that total lipid mass of white-fronts in the MAV increased from arrival in October to the highest values in November, and then continuously declined to the lowest levels in January and February. Furthermore, nearly all lipids accumulated in the MAV were exhausted prior to spring migration, potentially limiting the effectiveness of the MAV as an area to gain energy stores prior to spring migration. Thus, we hypothesize that white-fronts wintering in the MAV balance greater energy expenditure with greater energy income.

The MAV, Chenier Plain, and Texas Mid-coast are major rice producing regions, and white-fronts heavily utilize waste rice in both flooded and dry rice fields in these regions [48, 55], while other regions produce grain on dry land (e.g., corn, sorghum, peanuts) that do not require flooding. Geese that forage on the often highly available and easily extractable waste grains in dry agricultural fields can optimize intake rates and profitability compared to foraging in natural wetlands where foods such as tubers and seeds typically have comparably greater searching and handling times [7, 28]. Greater searching and handling time associated with natural wetland foods may be functionally similar to foraging in flooded rice fields where geese cannot use visual foraging and must rely on tactile techniques to locate food underwater, thus increasing time spent foraging. However, the energy content and digestibility of agricultural foods can vary greatly [61]. For instance, Alisauskas et al. [1] found that lesser snow geese (*Anser caerulescens*) foraging in rice fields would need to consume 4.3 times more food (dry mass) to provide the same daily energy intake compared to geese foraging on a corn based diet. This may partially explain greater time spent foraging and greater ODBA estimates by white-fronts in rice producing regions (Fig. 5).

Little information existed regarding the fidelity and movements of white-fronts among wintering regions, and breeding region specific distributions during winter. White-fronts used up to six distinct regions per winter, and conservation planning should consider the entire wintering range of white-fronts given their proclivity for large-scale movements. With regard to harvest management, the entire Midcontinent white-front breeding range is managed as one population [17]. Banding data indicate that harvest is having little to no effect on adult survival, which is static or increasing for specific breeding populations [25], yet harvest appeared to have a negative effect on adult survival in earlier decades on Interior Alaska breeding white-fronts [52]. Currently, > 50% of all recreational white-front harvest occurs in the south-central U.S. (i.e., Texas, Louisiana, and Arkansas [17];). Hunting-related disturbance may influence movements of white-fronts throughout their range. Indeed, daily white-front movements increased and peaked approximately 1 month after southern states opened hunting season for white-fronts, and decreased the remainder of the season. However, this effect was stronger in 2016–2017 than 2017–2018 (Fig. 2). Given we did not observe any spatial segregation of individuals from specific breeding areas among wintering regions, and white-fronts moved readily among wintering regions between flyways, states, and hunting zones, continuing white-front management at the Midcontinent scale seems appropriate.

White-fronts captured in four regions during winter subsequently summered across the entire previously-described breeding range, revealing a high degree of admixture on wintering areas among breeding affiliations and suggesting that large-scale movements occur at the population level and are not limited to segments of the population. Winter fidelity in pair-bonded geese is a male-driven trait, where males are believed to lead the female from breeding locations to wintering locations to which they are philopatric [65]. Here, 79 of 97 white-fronts used for movement analyses were female, and while we did not test for differences between sexes or pairing status, rates of movement among wintering regions were higher than expected regardless of this status, and may be greater for juvenile and unpaired individuals in the population [32, 73]. Considering males initiate pairing with females during winter, we may expect unpaired males to make regional movements at even larger rates than observed in this study. Wilson et al. [74] showed that the Midcontinent greater white-fronted goose population is panmictic across the Arctic breeding range. High rates of large-scale movements successfully mixes breeding regions during winter, which likely results in the pairing of individuals from different breeding regions. Therefore, movements during winter may determine population structure and gene flow within greater white-fronted goose subpopulations at the Midcontinent scale resulting in the observed panmictic population structure.

## Conclusions

Here we showed that rich location and behavior data can provide unprecedented complementary understanding of animal decision-making. Insights from these data can be particularly revealing for migratory birds that are only observed in a region for a portion of each annual cycle [16], in addition to non-migratory bird movements and behaviors among discrete habitat patches, center places (e.g., roosts) or breeding areas (e.g., leks). We anticipate that as tracking devices become increasingly miniaturized, the utility and diversity of these data to answer common questions in ecology and conservation will only increase [14]. In parallel to advancements in tracking technology, the statistical tools to analyze rich data sets are also improving. Hierarchical models parameterized in a Bayesian framework provide increasingly accessible opportunities for movement ecologists to include expert knowledge alongside collected data in a joint framework, and fully propagate uncertainty, to revise conservation plans for robust decision-making seasonally and annually, with increasingly limited financial resources. In this study, hierarchical multistate modeling revealed unprecedented intra-winter movement information and probable continuation of a winter distribution shift for Midcontinent Greater White-fronted Geese, that will aid future conservation planners in preparing for increasing winter abundances in some areas, and providing additional habitat resources for remaining areas with declining abundance. Together, the movement and behavior data from smaller devices and novel statistical tools are encouraging means for practitioners to tackle global challenges in climate and land use change [43].

## Supplementary Information

**Additional file 1.**

## Data Availability

The datasets used and/or analyzed during the current study are available from the corresponding author on reasonable request.

## Abbreviations

ACC: Accelerometer
CTT: Cellular tracking technologies
GPS: Global positioning system
GSM: Global system for mobile communication
MAV: Mississippi Alluvial Valley
ODBA: Overall dynamic body acceleration
