# Supplementary material for: Winter fidelity, movements, and energy expenditure of Midcontinent Greater White-fronted Geese
Source: Mov Ecol. 2021 Jan 20;9:2. doi: 10.1186/s40462-020-00236-4 (PMC7816378; doi:10.1186/s40462-020-00236-4)
Supplement: Supplementary file 1 — Additional file 1. [file 40462_2020_236_MOESM1_ESM.docx]

**Additional File 1**

**Winter Fidelity, Movements, and Energy Expenditure of Midcontinent Greater White-fronted Geese**

Jay A. VonBank, Mitch D. Weegman, Paul T. Link, Stephanie A. Cunningham, Kevin J. Kraai, Daniel P. Collins, and Bart M. Ballard

Additional Figure 1. Duration of GPS collection for each individual during winter 2016-2017 and 2017-2018. Gaps indicate missing GPS data or data censored to match duty cycles among transmitters.


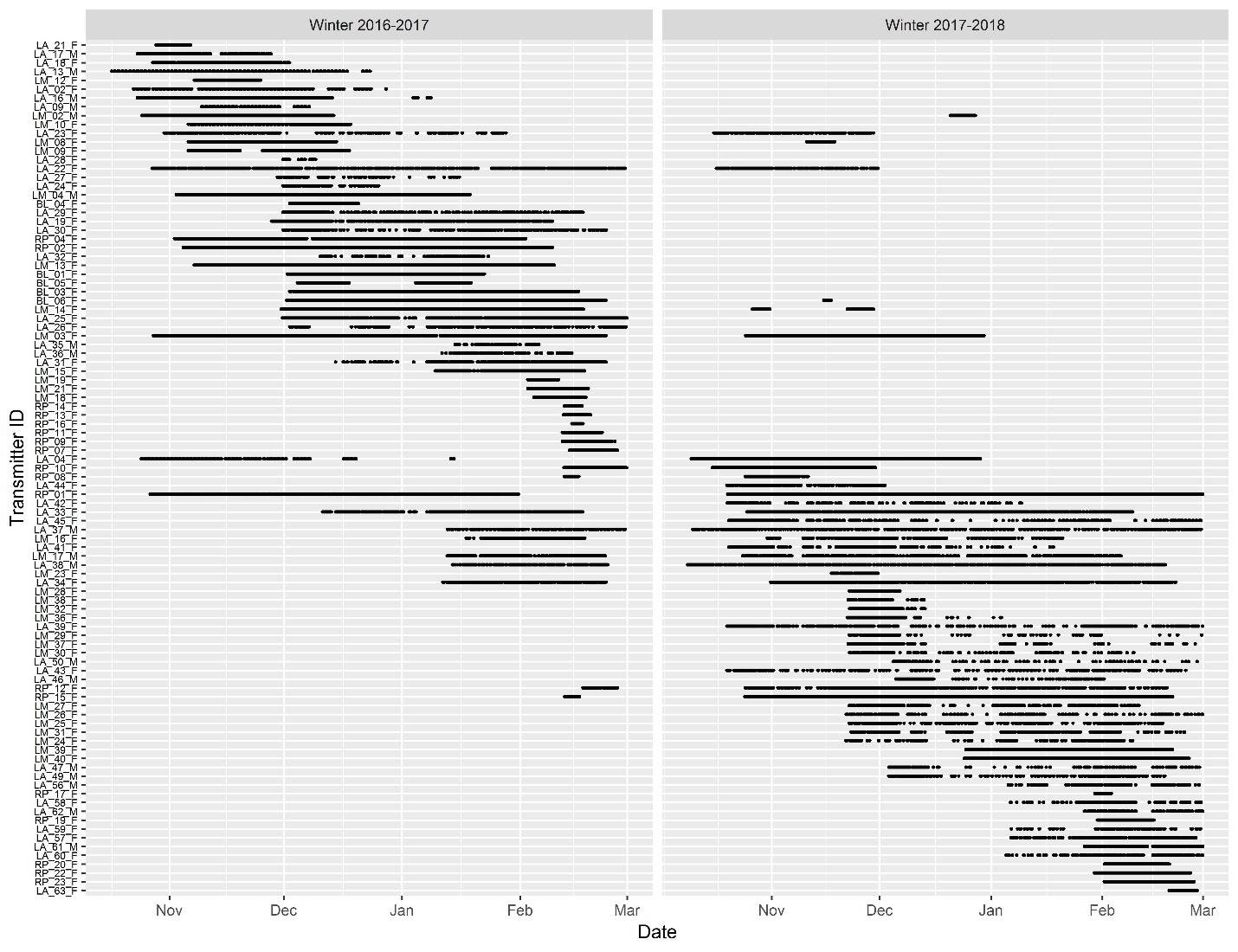


Additional Table 1. State transition matrix for multistate model to estimate regional transition probabilities of greater white-fronted geese (*Anser albifrons frontalis*) during winter.

| True State at Time *t* | True State at Time *t* + 1 | | | | | | | | |
| --- | --- | --- | --- | --- | --- | --- | --- | --- | --- |
| \|  \| \| --- \| | MAV | Chenier Plain | TX Mid-coast | Lower TX Coast | STX Brushlands | Rolling/High Plains | Mexico | Other | \| Not Seen \| \| --- \| |
| MAV (A) | *ϕ*_A_(1-*ψ*_AB_-*ψ*_AC_-*ψ*_AD_-*ψ*_AE_-*ψ*_AF_-*ψ*_AG_-*ψ*_AH_) | *ϕ*_A_*ψ*_AB_ | *ϕ*_A_*ψ*_AC_ | *ϕ*_A_*ψ*_AD_ | *ϕ*_A_*ψ*_AE_ | *ϕ*_A_*ψ*_AF_ | *ϕ*_A_*ψ*_AG_ | *ϕ*_A_*ψ*_AH_ | 1 - *ϕ*_A_ |
| Chenier Plain (B) | *ϕ*_B_*ψ*_BA_ | *ϕ*_B_(1-*ψ*_BA_-*ψ*_BC_-*ψ*_BD_-*ψ*_BE_-*ψ*_BF_-*ψ*_BG_-*ψ*_BH_) | *ϕ*_B_*ψ*_BC_ | *ϕ*_B_*ψ*_BD_ | *ϕ*_B_*ψ*_BE_ | *ϕ*_B_*ψ*_BF_ | *ϕ*_B_*ψ*_BG_ | *ϕ*_B_*ψ*_BH_ | 1 - *ϕ*_B_ |
| TX Mid-coast (C) | *ϕ*_C_*ψ*_CA_ | *ϕ*_C_*ψ*_CB_ | *ϕ*_C_(1-*ψ*_CA_-*ψ*_CB_-*ψ*_CD_-*ψ*_CE_-*ψ*_CF_-*ψ*_CG_-*ψ*_CH_) | *ϕ*_C_*ψ*_CD_ | *ϕ*_C_*ψ*_CE_ | *ϕ*_C_*ψ*_CF_ | *ϕ*_C_*ψ*_CG_ | *ϕ*_C_*ψ*_CH_ | 1 - *ϕ*_C_ |
| Lower TX Coast (D) | *ϕ*_D_*ψ*_DA_ | *ϕ*_D_*ψ*_DB_ | *ϕ*_D_*ψ*_DC_ | *ϕ*_D_(1-*ψ*_DA_-*ψ*_DB_-*ψ*_DC_-*ψ*_DE_-*ψ*_DF_-*ψ*_DG_-*ψ*_DH_) | *ϕ*_D_*ψ*_DE_ | *ϕ*_D_*ψ*_DF_ | *ϕ*_D_*ψ*_DG_ | *ϕ*_D_*ψ*_DH_ | 1 - *ϕ*_D_ |
| STX Brushlands (E) | *ϕ*_E_*ψ*_EA_ | *ϕ*_E_*ψ*_EB_ | *ϕ*_E_*ψ*_EC_ | *ϕ*_E_*ψ*_ED_ | *ϕ*_E_(1-*ψ*_EA_-*ψ*_EB_-*ψ*_EC_-*ψ*_ED_-*ψ*_EF_-*ψ*_EG_-*ψ*_EH_) | *ϕ*_E_*ψ*_EF_ | *ϕ*_E_*ψ*_EG_ | *ϕ*_E_*ψ*_EH_ | 1 - *ϕ*_E_ |
| Rolling/  High Plains (F) | *ϕ*_F_*ψ*_FA_ | *ϕ*_F_*ψ*_FB_ | *ϕ*_F_*ψ*_FC_ | *ϕ*_F_*ψ*_FD_ | *ϕ*_F_*ψ*_FE_ | *ϕ*_F_(1-*ψ*_FA_-*ψ*_FB_-*ψ*_FC_-*ψ*_FD_-*ψ*_FE_-*ψ*_FG_-*ψ*_FH_) | *ϕ*_F_*ψ*_FG_ | *ϕ*_F_*ψ*_FH_ | 1 - *ϕ*_F_ |
| Mexico (G) | *ϕ*_G_*ψ*_GA_ | *ϕ*_G_*ψ*_GB_ | *ϕ*_G_*ψ*_GC_ | *ϕ*_G_*ψ*_GD_ | *ϕ*_G_*ψ*_GE_ | *ϕ*_G_*ψ*_GF_ | *ϕ*_G_(1-*ψ*_GA_-*ψ*_GB_-*ψ*_GC_-*ψ*_GD_-*ψ*_GE_-*ψ*_GF_-*ψ*_GH_) | *ϕ*_G_*ψ*_GH_ | 1 - *ϕ*_G_ |
| Other (H) | *ϕ*_H_*ψ*_HA_ | *ϕ*_H_*ψ*_HB_ | *ϕ*_H_*ψ*_HC_ | *ϕ*_H_*ψ*_HD_ | *ϕ*_H_*ψ*_HE_ | *ϕ*_H_*ψ*_HF_ | *ϕ*_H_*ψ*_HG_ | *ϕ*_H_(1-*ψ*_HA_-*ψ*_HB_-*ψ*_HC_-*ψ*_HD_-*ψ*_HE_-*ψ*_HF_-*ψ*_HG_) | 1 - *ϕ*_H_ |
| Dead | 0 | 0 | 0 | 0 | 0 | 0 | 0 | 0 | 1 |

Additional Table 2. Observation matrix of true and observed states for multistate model to estimate regional transition probabilities of greater white-fronted geese (*Anser albifrons frontalis*) during winter.

| True State at Time *t* | True State at Time *t* + 1 | | | | | | | | |
| --- | --- | --- | --- | --- | --- | --- | --- | --- | --- |
|  | MAV | Chenier Plain | TX Mid-coast | Lower TX Coast | STX Brushlands | Rolling/High Plains | Mexico | Other | Not Seen |
| MAV (A) | \| *p*_A_ \| \| --- \| | 0 | 0 | 0 | 0 | 0 | 0 | 0 | \| 1 - *p*_A_ \| \| --- \| |
| Chenier Plain (B) | 0 | *p*_B_ | 0 | 0 | 0 | 0 | 0 | 0 | 1 - *p*_B_ |
| TX Mid-coast (C) | 0 | 0 | *p*_C_ | 0 | 0 | 0 | 0 | 0 | 1 - *p*_C_ |
| Lower TX Coast (D) | 0 | 0 | 0 | *p*_D_ | 0 | 0 | 0 | 0 | 1 - *p*_D_ |
| STX Brushlands (E) | 0 | 0 | 0 | 0 | *p*_E_ | 0 | 0 | 0 | 1 - *p*_E_ |
| Rolling/High Plains (F) | 0 | 0 | 0 | 0 | 0 | *p*_F_ | 0 | 0 | 1 - *p*_F_ |
| Mexico (G) | 0 | 0 | 0 | 0 | 0 | 0 | *p*_G_ | 0 | 1 - *p*_G_ |
| Other (H) | 0 | 0 | 0 | 0 | 0 | 0 | 0 | *p*_H_ | 1 - *p*_H_ |
| Dead | 0 | 0 | 0 | 0 | 0 | 0 | 0 | 0 | 1 |
